# 3D Virtual Reality Imaging of Major Aortopulmonary Collateral Arteries: A Novel Diagnostic Modality

**DOI:** 10.1177/21501351211045064

**Published:** 2021-11-23

**Authors:** Pieter C. van de Woestijne, Wouter Bakhuis, Amir H. Sadeghi, Jette J. Peek, Yannick J.H.J. Taverne, Ad J.J.C. Bogers

**Affiliations:** 1 Thoraxcenter, 6993 Erasmus University Medical Center, Rotterdam, the Netherlands

**Keywords:** congenital heart surgery, computer applications (includes simulation, artificial intelligence etc), imaging (all modalities), MAPCAs (major aortopulmonary collateral arteries), pediatric

## Abstract

**Background:**

Major aortopulmonary collateral arteries (MAPCAs), as seen in patients with pulmonary atresia, are arteries that supply blood from the aorta to the lungs and often require surgical intervention. To achieve complete repair in the least number of interventions, optimal imaging of the pulmonary arterial anatomy and MAPCAs is critical. 3D virtual reality (3D-VR) is a promising and upcoming new technology that could potentially ameliorate current imaging shortcomings.

**Methods:**

A retrospective, proof-of-concept study was performed of all operated patients with pulmonary atresia and MAPCAs at our center between 2010 and 2020 with a preoperative computed tomography (CT) scan. CT images were reviewed by two congenital cardiac surgeons in 3D-VR to determine additional value of VR for MAPCA imaging compared to conventional CT and for preoperative planning of MAPCA repair.

**Results:**

3D-VR visualizations were reconstructed from CT scans of seven newborns where the enhanced topographic anatomy resulted in improved visualization of MAPCA. In addition, surgical planning was improved since new observations or different preoperative plans were apparent in 4 out of 7 cases. After the initial setup, VR software and hardware was reported to be easy and intuitive to use.

**Conclusions:**

This study showed technical feasibility of 3D-VR reconstruction of children with immersive visualization of topographic anatomy in an easy-to-use format leading to an improved surgical planning of MAPCA surgery. Future prospective studies are required to investigate the clinical benefits in larger populations.

## Introduction

In pulmonary atresia with ventricular septal defect (PA-VSD), major aortopulmonary collateral arteries (MAPCAs) are often present. These MAPCAs can be essential in patients with diminutive pulmonary vascular abnormalities.^1^ As a consequence, lung areas can be vascularized via single, noncommunicating blood supply (MAPCAs only) or via dual, communicating blood supply (both MAPCA and main pulmonary artery [MPA]).^2^ If adequate antegrade pulmonary arterial circulation is present, dual-supply MAPCAs can be occluded (surgically or therapeutically), while MAPCAs with single lung segment perfusion to the pulmonary circulation should be relocated from the aorta to the native pulmonary arterial system (unifocalization procedure).^3^

The goal of surgical correction is to eventually create a biventricular circulation with an adequate pulmonary arterial system, to which all MAPCAs with singular blood supply are unifocalized and in which the dual-supply MAPCAs are ligated, preferably in the least number of operations. Due to patient-specific (under)-development of the pulmonary arterial system and variance in the number of MAPCAs, systemic-to-pulmonary artery (PA) shunt placement or right ventricular outflow tract (RVOT) procedure to support pulmonary arterial outgrowth and/or multistage MAPCA unifocalization may be necessary.^4,5^ Unifocalization may be a complex surgical procedure and can be associated with increased morbidity and mortality.^6^ Nevertheless, recent studies report improved survival by a higher percentage of complete repair and fewer operations per patient.^7^

Complete repair with a limited number of interventions depends on optimal visualization of the pulmonary arterial anatomy and MAPCAs and precise preoperative planning. Currently, in our center, preoperative planning is routinely performed with catheterization angiography (CA) and computed tomography angiography (CTA) where the emphasis on three-dimensional (3D) topographical anatomical relationships is sometimes lacking. Furthermore, two-dimensional (2D) CA and CTA imaging require an advanced level of 3D imaging review expertise on the part of the cardiac surgeon and cardiovascular radiologist. Previous studies have shown that identification of anatomical structures and their course is superior in 3D visualization compared with 2D.^8^ 3D volume rendering of CT scans is a novel technology, and can be time-consuming and requires extra resources such as dedicated imaging software, a radiologist, or radiology technician.^9^ In addition to this, 3D-CT images are still shown on a flat, 2D computer screen, which lacks depth perception and interaction.^10^

3D-Virtual Reality (3D-VR) visualization is a promising and innovative new technology that could potentially complement the shortcomings of 2D imaging.^11^ Recent studies, including publications by our group, have shown that VR can provide true in-depth perception, intuitive model manipulation and interaction, structure segmentation, and can enable turning on/off surrounding structures during the assessment^12–14^. In other surgical departments, 3D-VR surgical planning has been shown to reduce operation duration and decrease the length of stay in hospital.^15^

In this retrospective single-center pilot study, we aimed to study the technical feasibility, clinical usefulness, and experience of multiple users on the application of 3D-VR for the preoperative planning of patients with PA-VSD and MAPCAs.

## Patients and Methods

### Patient Selection

Seven patients diagnosed with PA-VSD with MAPCAs who underwent corrective repair between 2010 and 2020 at our center were eligible for inclusion since both preoperative CTA and CA images were available. An overview of the baseline characteristics is provided in Table 1. Median age at time of CTA was 2 days (range 2-388 days). CA was planned in the week before surgery, median age 213 days (range 29-506 days). The study was approved by the local medical ethical committee (MEC-2020-0891) of Erasmus University Medical Center. Informed consent was obtained from the legal representatives of all living patients before inclusion.

**Table 1.** Patient Characteristics.

Pat #	Gender	Age at CT Scan (days)	Age at CA (days)	Patient's Anatomy	Performed Surgery	Additional Value of 3D-VR over 2D-CT
1	M	301	42	Two MAPCAs branching off the descending aorta, one to left lung, other to right lung.	(1) Central shunt	Better visualization of MAPCAs dorsally and ventrally of the esophagus.
2	F	2	213	Two MAPCAs from descending aorta to right upper lobe and left lower lobe.	(1) mBT shunt	Stenosis of the left PA was suggested in 3D-VR, forecasting a decreased effect of central shunt placement. In retrospect, central shunt placement on such small LPA should be reconsidered.
					(2) Unifocalization left + mBT shunt	
					(3) Unifocalization right.	
					(4) Complete RVOT reconstruction (allograft).	
3	M	2	29	Four MAPCAs in total sprouting from descending aorta; two large arteries (one to right PA, other to left PA) and two small arteries to left lower lobe.	(1) Melbourne shunt	No evident additional value over 2D-CT.
					(2) Unifocalization right + mBT shunt	
					(3) Unifocalization left + mBT shunt.	
4	M	2	127	Three MAPCAs, two to all right lung lobes, and one to left lobes.	(1) Melbourne shunt.	Tortuosity of MAPCAs, in relation to surrounding structures, was better visualized in 3D-VR.
					(2) mBT shunt.	
					(3) mBT shunt left + unsuccessful unifocalization left.	
					(4) Complete RVOT reconstruction (allograft) + unifocalization right.	
5	M	3	252	Two large MAPCAs from proximal descending aorta to both lungs.	(1) Melbourne shunt	Offspring and branching of MAPCAs was made clearer for the surgeon in 3D-VR than in 2D-CT.
					(2) Unifocalization right + mBT shunt	
					(3) Unifocalization left + mBT shunt (4) MPA augmentation	
					+ closing Melbourne shunt & mBT left.	
					(5) Complete RVOT reconstruction (allograft).	
6	F	2	223	Three MAPCAs, distal aortic arch to both upper lobes, proximal descending aorta to right lower lobe and descending aorta to left lower lobe.	(1) Central shunt	Course of MAPCA in relationship to PA; although intrapulmonary connection was suggested, it could not
					(2) Unifocalization right + mBT shunt.	
					(3) Unifocalization left.	be confirmed by CA.
					(4) Complete RVOT reconstruction (allograft)	
7	M	388	506	Distally from isthmus a large MAPCA to left lung hilum	(1) mBT shunt right.	3D-VR showed that mBT shunt was placed between aorta and MAPCA, instead on right PA. This had not been noticed on CA or 2D-CT.
					(2) Unifocalization left + mBT shunt	
					(3) Melbourne shunt.	
					(4) Complete RVOT reconstruction (allograft).	

All performed cardiothoracic interventions to date are shown chronologically. Complete RVOT reconstruction includes VSD closure, closing of all (remaining) shunts and RV-PA reconstruction.

CA: catheterization angiography, CT: computed tomography, F: female, M: male, MAPCA: major aortopulmonary collateral artery, mBT: modified Blalock-Taussig, MPA: main pulmonary artery, PA: pulmonary artery, RLL: right lower lobe, RUL: right upper lobe, RVOT: right ventricle outflow tract, VR: virtual reality.

### Computed Tomography in 3D, Image Segmentation, and Virtual Reality Rendering

To create volumetric 3D-VR rendering of the CT scans, preoperative CTA scans were used (Supplemental Material A). There were no specific technical requirements for CT scans and all scans were performed at our center according to the local protocol. Semiautomatic 3D segmentation was performed to highlight anatomical structures (eg bronchi, PA, and MAPCAs) by using a previously published protocol.^16^ Digital imaging and communications in medicine (DICOM) files and corresponding 3D segmentation files were loaded into our CardioVR software (MedicalVR, Amsterdam, the Netherlands), which creates an automatic 3D-VR visualization. Afterwards, the segmentation was checked by an experienced physician. 3D-VR was directly available for the user by using a VR head-mounted display and associated controllers.

### Study Design, Objectives, and Data Analysis

Within this pilot study, our objective was to study the technical feasibility, clinical usefulness, and user experience of 3D-VR for preoperative planning of newborns with PA-VSD with MAPCAs. Technical feasibility was defined as the possibility to create and immersively review 3D reconstructions of (low-resolution) CTA scans of newborns in VR. Clinical usefulness, the additional value of 3D-VR over 2D-CT, was determined via (1) if specific characteristics were found with 3D-VR and (2) if surgical preoperative planning would be altered after 3D-VR reconstruction. Thirdly, the user experience of 3D-VR environment was evaluated via a nonstandardized, written questionnaire (Supplemental Material B).

Two experienced congenital cardiothoracic surgeons were included as study participants. A brief (10 min) audiovisual hardware and software instruction was provided. During the assessments, an experienced VR user was on site for technical support. Both participants reviewed all imaging modalities (CA, 2D-CT, and 3D-VR) individually per case and described various MAPCA-related parameters, after which a surgical plan was drafted. The participants were blinded to each other's descriptions and surgical plans. Original surgery reports were obtained from the electronic health record to compare the surgeons’ devised plan.

### Data Visualization and Analysis

2D computer screen recordings of the 3D-VR environment were captured and stills of this video were used for figures. Additional 3D model reconstructions, only used for visualization, were made in 3D Slicer.^17^ All data was analyzed using Microsoft Office Excel (Microsoft).

## Results

### Technical Feasibility

**Figure 1.**
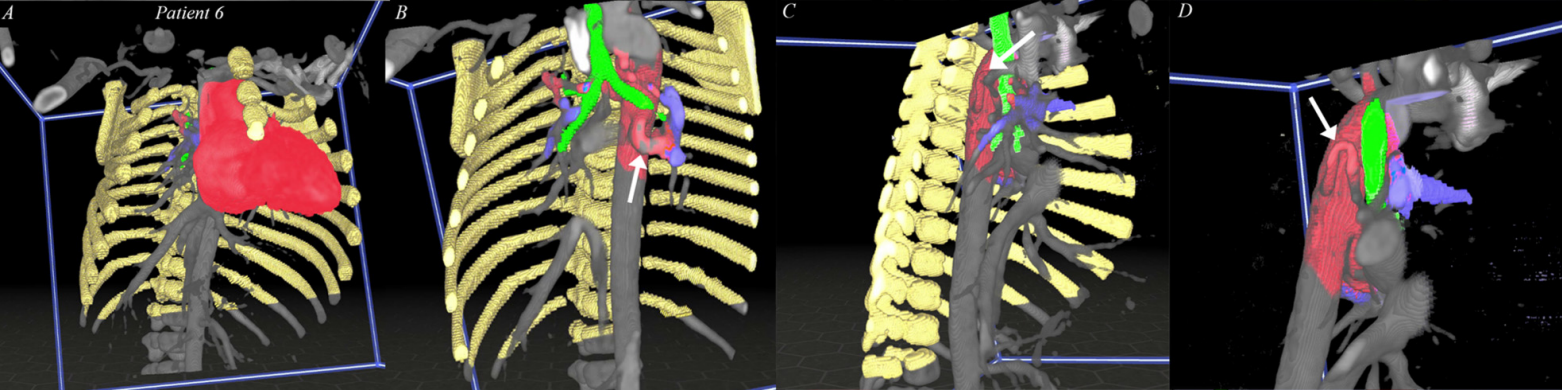
*Realtime 3D-VR visualization of segmented CT scan*. (A) structures can be segmented, colored and visualized as an overlay over the grayscale CT scan. Rib cage & sternum (yellow), heart (red), bronchus (green), PA (purple). (B) Movable transection panel can be placed in the model, to visualize the structures of interest. Aorta and MAPCA (red, pointed by white arrow). (C) Rotation of the model, in cooperation with the movable transection plane, to provide better overview of MAPCA and PA. (D) All irrelevant structures (rib cage, heart, etc) can be hidden, providing better and more zoomed-in view of offspring and course of MAPCA. CT: computed tomography, PA: pulmonary artery, MAPCA*:* major aortopulmonary collateral artery.

**Figure 2.**
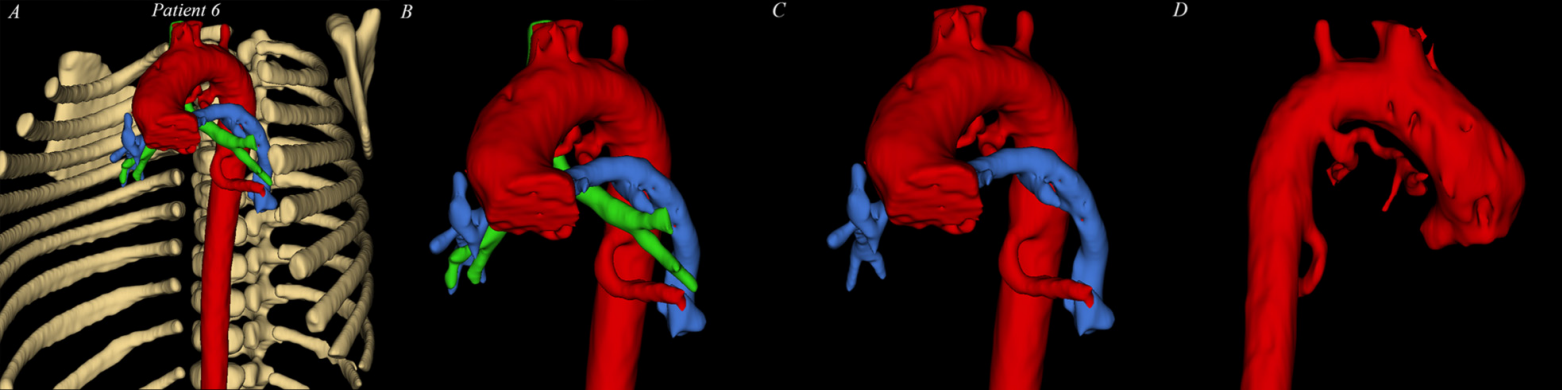
*3D-VR Segmentation visualization in 3D slicer*. (A) complete segmentation of patient 6: rib cage (white), aorta + MAPCAs (red), bronchus (green), PA (blue). (B) zoomed in to show relationship between MAPCA, trachea, and PA. (C & D) Inactivating bronchus and PA segmentation to show the offspring and course of MAPCAs more precisely. PA: pulmonary artery. MAPCA: major aortopulmonary collateral artery, VR: virtual reality.

It was technically feasible to create 3D reconstructions of segmented CTA scans of all 7 newborns in VR (Figures 1 and 2, animated GIF figure in Supplemental Material C). A total of 6 scans had adequate spatial resolution and contrast enhancement for VR imaging, but CT scan and consequently the VR experience of patient 2 was suboptimal. Segmentation of the esophagus, which provides useful information about the course of MAPCAs around this structure, was possible in f4 patients.

### Clinical Usefulness

**Figure 3.**
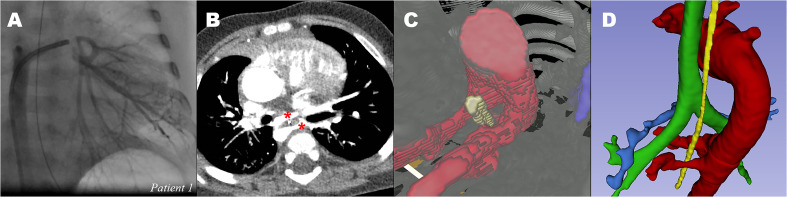
*Major aortopulmonary collateral arteries around esophagus of patient 1*. (A) CA of MAPCAs of interest to the left lung. (B) computed tomogram showing two MAPCAs (red *) to the left lung with esophagus in-between. (C) 3D-VR visualization of MAPCAs (red) and esophagus (yellow). (D) 3D segmented image to show aorta and MAPCA (red), esophagus (yellow), trachea (green) and native pulmonary artery (blue). CA: catheterization angiography, CT: computed tomography, MAPCA: major aortopulmonary collateral artery. *indicate two MAPCAs on the 2D-CT image, and are visualized in 3C and 3D in the 3D models. (See full color figure in online version of this article)

**Figure 4.**
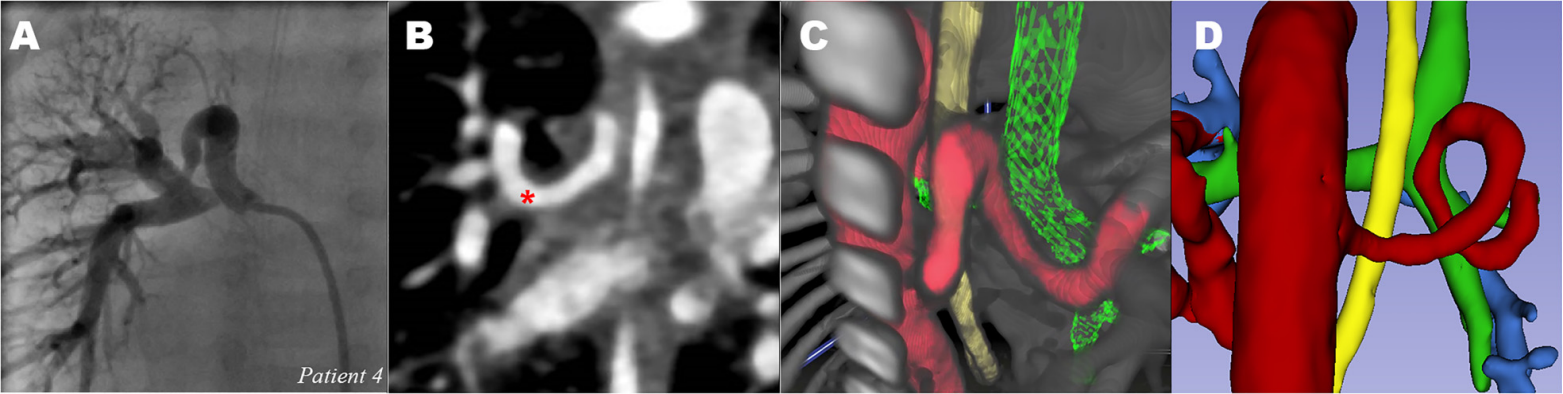
*Tortuous MAPCA: major aortopulmonary collateral artery of patient 4.* (A) CA of MAPCA to right lung (B) CT showing spiral course of MAPCA to right lung (red *). (C) 3D-VR visualization of MAPCAs (red), viewed from dorsal, showing tortuous course. Trachea (green) and esophagus (yellow). (D) 3D segmented image showing tortuous MAPCA from dorsal. No connection with pulmonary artery (blue). CA: catheterization angiography, CT: computed tomography, MAPCA: major aortopulmonary collateral artery; 3D-VR: 3D virtual reality. *indicate two MAPCAs on the 2D-CT image, and are visualized in 3C and 3D in the 3D models. (See full color figure in online version of this article)

**Figure 5.**
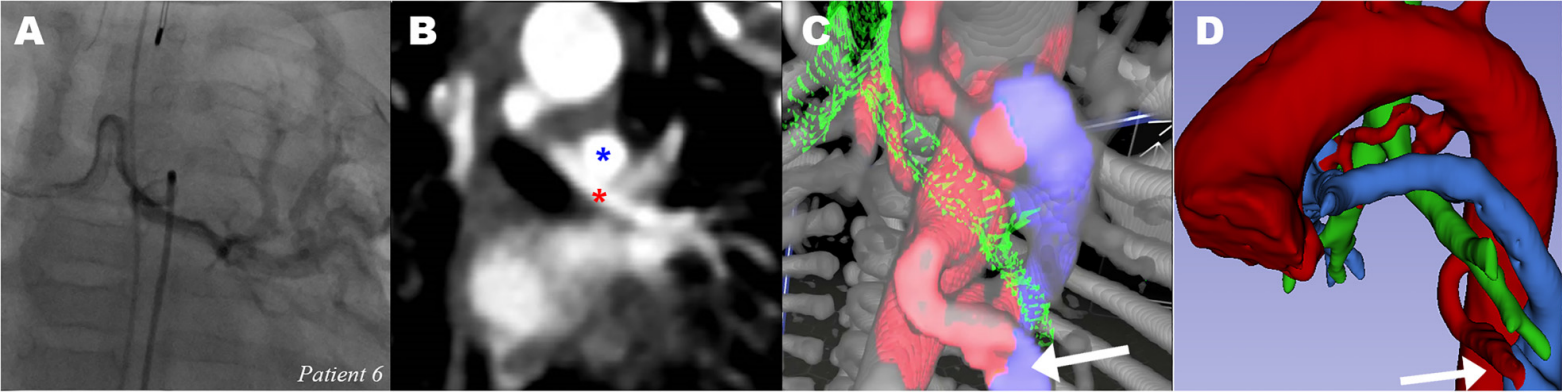
*Communicating major aortopulmonary collateral arteries of patient 6* (A) CA of MAPCA to left lung (B) CT showing MAPCA to left lung (red *) and left PA on top (blue *). (C) 3D-VR visualization of MAPCAs (red), suggesting communication (white arrow) between lower MAPCA (red) and PA (blue). bronchus (green). (D) 3D segmented image with suggested intrapulmonary connection (white arrow). CA: catheterization angiography, CT: computed tomography, PA: pulmonary artery, MAPCA: major aortopulmonary collateral artery; 3D-VR, 3D virtual reality. *indicate two MAPCAs on the 2D-CT image, and are visualized in 3C and 3D in the 3D models. (See full color figure in online version of this article)

**Figure 6.**
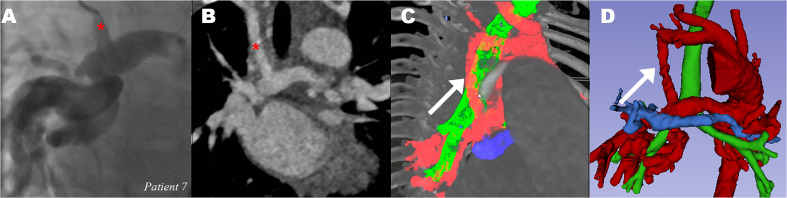
*Modified Blalock-Taussig shunt from right subclavian artery to major aortopulmonary collateral artery of patient 7*. (A) CA of catheter in mBT shunt (red *) (B) CT showing BT shunt to MAPCA (red *) (C) 3D-VR visualization of MAPCAs (red), mBT shunt pointed out by white arrow. Bronchus (green) and PA (blue). (D) 3D Slicer image clearly shows BT shunt on MAPCA (white arrow) instead on right PA (blue). *CA: catheterization angiography, CT: computed tomography, mBT: modified Blalock-Taussig, PA: pulmonary artery, MAPCA: major aortopulmonary collateral artery.* *indicate two MAPCAs on the 2D-CT image, and are visualized in 3C and 3D in the 3D models. (See full color figure in online version of this article)

The usefulness and additional value of 3D-VR over 2D-CT is shown in Table 1. An extensive description of all patient's CA, 2D-CT, and 3D-VR by both surgeons is presented in Supplemental Material D. All MAPCAs could be segmented and visualized by the software, except 2 very small MAPCAs in patient 4 and 1 small MAPCA in patient 6 of 1 mm in diameter. A total of 4 cases are featured since these cases showed remarkable characteristics. In patient 1, at almost 1 year old, branching of the proximal MAPCA around the esophagus was clearly visualized in 3D-VR (Figure 3). In patient 4, a 6 month-old male, the tortuous course of the right MAPCA behind the trachea was clearly visible (Figure 4). 3D-VR of patient 6 suggested intrapulmonary connections of both MAPCAs to the left (to left upper lobe [LUL] and left lower lobe [LLL]) with the native PA circulation (Figure 5). However, CA showed only one communicating MAPCA (to LLL) (Figure 5A). During surgery, no intrapulmonary connection of the left lung was seen. 3D-VR visualization of patient 7 showed that the modified Blalock–Taussig (mBT) shunt had been placed onto the MAPCA (Figure 6). This anastomosis had not been noticed after CA and 2D-CT review at time of reporting by cardiac surgeon or by an experienced radiologist (Figure 6C).

### VR User Experience

Questionnaire scores are shown in Table 2. Both surgeons found the VR environment easy to use, and the VR model easy to interact with. Although VR assessment takes more time (∼15–20 min) than conventional imaging techniques, mainly due to setting up the VR hardware and software, the 3D-VR model was of additional value to assess the number of MAPCAs, the offspring of MAPCAs and the course of MAPCAs. Classification of (non-)communicating arteries scored low. Lastly, the threshold to use VR is still too high due to the inability to load the images in the VR computer by surgeons themselves. The future perspective to become standard image modality is still unknown. More comments on the VR simulation are included in Supplemental Material E.

**Table 2.** Questionnaire Outcomes.

	Surgeon 1	Surgeon 2
The VR environment is easy to use.	4 of 5	4 of 5
The assessment of VR reconstruction takes more time than conventional imaging techniques.	2 of 5	4 of 5
Interaction with the reconstruction (ie turning/coloring/slicing) is easy to learn and to use during assessment.	4 of 5	4 of 5
Interaction with the reconstruction (ie turning/coloring/slicing) is of additional value for understanding MAPCA pathology.	3 of 5	5 of 5
This model is of additional value to the current imaging techniques in assessing the number of MAPCAs.	3 of 5	4 of 5
This model is of additional value to the current imaging techniques in assessing the offspring of the MAPCAs from the aorta.	5 of 5	5 of 5
This model is of additional value to the current imaging techniques in assessing the course of the MAPCAs	3 of 5	5 of 5
This model is of additional value to the current imaging techniques in the classification of (non)communicating arteries.	2 of 5	2 of 5
This model is of additional value to the current imaging techniques in preoperative planning	4 of 5	4 of 5
The threshold to use VR is still too high for me (given the required hardware, software and time to load)	4 of 5	1 of 5
I think VR reconstruction will become a standard image modality in preoperative planning for congenital heart surgery.	3 of 5	3 of 5

Average score of questionnaires by both surgeons. Scored from 1 to 5 stars, from totally disagree (1) to totally agree (5). Scores were rounded down and visualized by black stars.

VR: virtual reality, MAPCA: major aortopulmonary collateral artery.

## Comment

This study showed that 3D-VR for imaging review and preoperative planning of newborns with PA-VSD-MAPCAs was possible and addressed the fact that, albeit still time-consuming, surgeons can retrieve important patient-specific details to improve surgical procedure, with an excellent user experience. 3D-VR reconstruction of CTA scans could be created of all newborns, which is more difficult than in adults due to smaller patient dimensions and suboptimal contrast enhancement. 3D-VR can be used as a complementary modality for preoperative planning of MAPCA repair to provide better overview of the structures of interest for the surgeon. The VR environment was easy to use by both surgeons and had additional value in the assessment of number, origin, and course of MAPCAs. Presence of a nasogastric tube is a recommendation, due to possible segmentation of the esophagus and visualization of the course of the MAPCAs in relation to the esophagus in 3D-VR.

The less optimal CT scan and VR experience of patient 2 could be explained by a thicker slice width and contrast timing. The approach of scanning newborns with congenital heart disease in our center is manually triggered CT scanning due to aberrant and patient-specific anatomy, especially with MAPCAs, plus combining pulmonary and systemic arterial phase to lower total radiation dose (3 cc/kg contrast), which is currently suboptimal for VR purposes. Because of this suboptimal contrast injection, suggestion of intrapulmonary connection between MAPCAs and native pulmonary system based on the CT scan (either 2D-CT or 3D-VR) should be viewed with suspicion, as a false-positive connection was observed in patient 6. CA remains a golden standard to determine single or dual supply.

All patients included in our study were treated with a staged approach to reach corrective surgery. Midline one-stage complete unifocalization claims to lower potential adverse consequences compared to a staged approach (ie increased perioperative risks due to multiple hospitalizations and operations).^18,19^ This is the case despite single-stage unifocalization being a more complex operation, with risks including incomplete repair.^18,19^ Due to the latter, midline one-stage unifocalization has not (yet) been performed in our center. 3D-VR could possibly, via more optimal MAPCA visualization, contribute to a higher percentage of complete repair in both approaches and could potentially facilitate one-stage midline repair in some patients. Despite this, MAPCA repair (choice of approach and unifocalization/ligation) will remain difficult, due to the variation in pulmonary arterial and MAPCA anatomy as well as method of repair due to differences in experience and approaches worldwide.

3D-VR is, after an initial cost for essential software and hardware components, readily available and offers a low-cost method to visualize specific anatomic structures. Costs for “off the shelf” hardware, consisting of a high-performance computer and VR devices, starts at $1500 to 2500. Disadvantages of other visualization techniques for preoperative planning, such as 3D printing, are that these models are static and lack interaction and multiangle sliced views, without information about surrounding structures, and require printing time and material.^20,21^

This proof-of-concept study showed the additional value of implementation of 3D-VR imaging in the preoperative planning of MAPCA repair, which assists surgeons with the difficult unifocalization procedure. The retrospective character of this study is a limitation, since we could not obtain real-life information about preoperative planning nor real-life feedback about the MAPCA segmentation in 3D-VR, although surgical reports were available. A prospective case series would be valuable to overcome this limitation.

### Future Perspectives

In the future, we are planning to study the implementation of 3D-VR for various (congenital) heart diseases, and to collaborate with multiple medical centers. Furthermore, in the near future, we hope to be able to provide detailed information about blood supply to specific lung segments and classify intrapulmonary communications, based on the 3D-VR visualization. This feature is already available in adults, but spatial resolution and contrast enhancement in pediatric cardiac patients is still inadequate to visualize bronchial segmental branches and specific pulmonary segmental arteries. Implementing additional image modalities in our VR environment, such as CA or even rotational angiography (RA) or magnetic resonance angiography (MRA), is another future goal, which may comprise the information of both CTA and CA and result in fewer scans necessary and lower radiation exposure.^22,23^ However, RA and MRA are still under development and not (yet) performed in our center.^22,23^ Lastly, we are exploring the possibilities of augmented reality for visualization and segmentation as perioperative navigation, which is already used in hepatobiliary surgery, since perioperative guidance can help surgeons to find the origin and course of multiple MAPCAs without the need to rely on their memory of preoperative images.^24^

## Supplemental Material

sj-docx-1-pch-10.1177_21501351211045064 - Supplemental material for 3D Virtual Reality Imaging of Major Aortopulmonary Collateral Arteries: A Novel Diagnostic Modality Click here for additional data file. Supplemental material, sj-docx-1-pch-10.1177_21501351211045064 for 3D Virtual Reality Imaging of Major Aortopulmonary Collateral Arteries: A Novel Diagnostic Modality by Pieter C. van de Woestijne, Wouter Bakhuis, Amir H. Sadeghi, Jette J. Peek, Yannick J.H.J. Taverne and Ad J.J.C. Bogers in World Journal for Pediatric and Congenital Heart Surgery

## Abbreviations and Acronyms

2D: Two-dimensional
3D: Three-dimensional
3D-VR: Three-dimensional virtual reality
CA: Catheterization angiography
CT: Computed tomography
CTA: Computed tomography angiography
DICOM: Digital imaging and communications in medicine
HMD: Head mount display
LUL: Left upper lobe
LLL: Left lower lobe
MAPCA: Major aortopulmonary artery
mBT: Modified Blalock–Taussig
MPA: Main pulmonary artery
MRA: Magnetic resonance angiography
PA: Pulmonary artery
PA–VSD: Pulmonary atresia with ventricular septal defect
RA: Rotational angiography
RLL: Right lower lobe
RUL: Right upper lobe
RVOT: Right ventricle outflow tract
