# Supplementary material for: 3D Virtual Reality Imaging of Major Aortopulmonary Collateral Arteries: A Novel Diagnostic Modality
Source: World J Pediatr Congenit Heart Surg. 2021 Nov 23;12(6):765–72. doi: 10.1177/21501351211045064 (PMC8637380; doi:10.1177/21501351211045064)

**Supplemental Material**

**3D Virtual Reality Imaging of Major Aortopulmonary Collateral Arteries: A Novel Diagnostic Modality**

Pieter C. van de Woestijne*, MD^1^, Wouter Bakhuis*, MD, MSc^1^, Amir H. Sadeghi, MD, MSc^1^, Jette J. Peek, BSc^1^, Yannick J.H.J. Taverne, MD, PhD^1^, Ad J.J.C. Bogers, MD, PhD^1^

^1^Department of Cardiothoracic Surgery, Thoraxcenter, Erasmus University Medical Center, Rotterdam, The Netherlands.

**A.** *VR-rendering workflow*

(A) CT scan is downloaded, anonymized and (B) structures of interest are semi-automatically segmented. (C) Both CT and segmentation files are loaded into our VR hardware & software. (D) Visualization is checked by surgical resident and is (E) ready to view with HMD by the surgeon.

*CT: computed tomography, HMD: head-mount-display, VR: virtual reality.*

**B. Questionnaire: VR reconstruction: user friendliness & additional value**

| Question | Strongly disagree – strongly agree |
| --- | --- |
| The VR environment is easy to use. | 1-2-3-4-5 |
| The assessment of VR reconstruction takes more time than conventional imaging techniques. | 1-2-3-4-5 |
| Interaction with the reconstruction (ie turning/coloring/slicing) is easy to learn and to use during assessment. | 1-2-3-4-5 |
| Interaction with the reconstruction (ie turning/coloring/slicing) is of additional value for understanding MAPCA pathology. | 1-2-3-4-5 |
|  |  |
| This model is of additional value to the current imaging techniques in assessing the number of MAPCAs | 1-2-3-4-5 |
| This model is of additional value to the current imaging techniques in assessing the offspring of the MAPCAs from the aorta. | 1-2-3-4-5 |
| This model is of additional value to the current imaging techniques in assessing the course of the MAPCAs | 1-2-3-4-5 |
| This model is of additional value to the current imaging techniques in the classification of (non-)communicating arteries. | 1-2-3-4-5 |
| This model is of additional value to the current imaging techniques in pre-operative planning | 1-2-3-4-5 |
|  |  |
| The threshold to use VR is still too high for me (given the required hardware, software and time to load) | 1-2-3-4-5 |
| I think VR reconstruction will become a standard image modality in preoperative planning for congenital heart surgery | 1-2-3-4-5 |

Comments on VR reconstruction: …

1. What is your opinion about the implementation of VR reconstruction for pre-operative planning?
2. What is your opinion about the use of VR reconstruction during surgery?

**C. Animated GIF figure of patient 6**

Rotating image of patient 6, with aorta and MAPCA colored in red, bronchi in green and native pulmonary system in blue. First, the CT scan is projecting over the segmentation files. Halfway through the animation, the grayscale CT scan is turned off, after which only the segmentation files are visible.

**D. CA, CT and 3D-VR description**

If only one description of the MAPCA based on the specific image modality is written down, this means that the observations of both surgeons agreed. In case of a different view, it is specifically mentioned.

*Patient 1*

**CA** showed two MAPCAs, both located at the descending aorta. The first bifurcates proximally into one right lung branch and one left lung branch with dual supply. The second MAPCA supplies the right lung, and has dual supply as well. Pulmonary confluence is very slender. **CT scan** showed a right descending aortic arch, medially located MAPCA, splitting into left and right branch, and the second MAPCA with a lateral offspring, to LLL. **VR** showed that MAPCAs are located very dorsal in the thorax, surrounded by trachea and PA, difficult to reach due to the right arch. The MAPCAs to the left are both dorsal and ventral to the esophagus. Native pulmonary artery is very small, especially RPA*.*

*Patient 2*

**CA,** which is performed after central shunt placement, showed stenosis of the left pulmonary artery. Two MAPCAs were seen, one to the right lung with dual supply without RUL. The second MAPCA has a small diameter and perfuses the left lung, with questionable dual supply. **CT scan** was poor, with low contrast and resolution. The MAPCA to the left could not be visualized. The MAPCA to the right lung was clearly visible. As a consequence of the CT scan, the **VR** quality was bad, with low spatial resolution. The second MAPCA could not be visualized as well. MAPCA to the right lung was segmented and visualized. Patent ductus arteriosus to native pulmonary confluence did already show stenosis of the LPA, which can forecast decreased effect of the central shunt placed two days later.

*Patient 3*

Three MAPCAs were visualized with **CA**. One MAPCA provides the entire left lung. The second, smaller MAPCA perfuses the RUL. At last, the third MAPCA provides the RLL. PA was not visualized. **CT** showed all three MAPCAs and very small PA. VR reconstruction showed one large MAPCA to RML and RLL. Visually, a connection between the MAPCA and LPA or proximal to the LUL artery is suggested. A small MAPCA to the RUL is located dorsally and courses behind the esophagus. Native PA branches are very small and were almost invisible in **VR** due to poor segmentation.

*Patient 4*

**CA** was performed after central shunt placement; stenosis of the RPA was visible. Two large MAPCAs were visualized, the first providing RUL, and the second the LLL, both communicating. **CT** showed two large MAPCAs as well. The first provided the right lung, with suggestion of connection in the pulmonary hilus. The other, perfusing the left lung, communicated with LPA. In addition, two very small MAPCAs (1mm diameter) were visualized, both towards the LLL. **VR** showed the two large MAPCAs, which both seemed to have a connection with the native circulation. The tortuous course of the right MAPCA was clearly visible and went behind the trachea, resulting in poor accessibility; only in the pulmonary hilus, not at the origin. Small MAPCAs could not be visualized.

*Patient 5*

**CA** performed after shunt placement, which showed RPA stenosis. Two MAPCAs without dual supply were visualized by one surgeon, to RUL, and the second to the left lung and RLL. The second surgeon saw four individual MAPCAs, two to RUL, one to left lung and one to RLL. **CT scan** showed however that two large MAPCAs are located at the ventral side of the descending aorta and bifurcate very proximal (one MAPCA with two branches to the right, second MAPCA with one branch to the left lung, other to the right lung). MPA and PA branches were very gracile. **VR** showed very small PA branches, that could not be traced peripherally. Esophagus could not be visualized due to missing nasal gastric tube. Two large, tortuous MAPCAs could be visualized and followed. Destinations corresponded with CA and CT.

*Patient 6*

**CA** was performed after Melbourne shunt placement. The first MAPCA showed connection with the RML and RLL, without dual supply. The second perfused LUL and RUL, also without dual supply. Third MAPCA to LLL communicated with native PA. **CT** showed a MAPCA with its offspring on the inner side of the aortic arch, branching to both LUL and RUL. MAPCA ventral of the descending artery provides the LLL. At last, a very small MAPCA (1mm diameter) goes to the RLL. The native MPA and PA branches are relatively small, 3-4mm in diameter. **VR** showed both MAPCAs, with presumably intrapulmonary connection between both left branching MAPCAs and the native pulmonary circulation. MAPCA to the RLL was not visualized.

*Patient 7*

**CA,** after modified BT shunt placement, showed a large MAPCA to the left lung with two branches and dual supply. In addition, it showed a connection between the pulmonary circulation and descending aorta, presumably ductus arteriosus. **CT scan** showed PA with reasonable diameter. MAPCA to the left forks into two branches, suppling the left lung. **VR** showed that the BT shunt was placed on the MAPCA. The large MAPCA to the left communicated with native PA, visualized by segmentation.

**E. Additional positive (+) arguments, improvements (-) and suggestions (o) on the VR simulation by both surgeons via the questionnaire**

+ Additional value of understanding MAPCA pathology highly depends on CT-scan quality and the quality of the segmentation. The offspring and course of the MAPCA is greatly shown, but unfortunately, sometimes the MAPCA cannot be tracked distally.

+ Additional value is high in assessing the course of MAPCAs, especially in relationship to the esophagus and trachea.

+ Diameters of structures, for example PA, in relationship to MAPCAs and/or shunts is easier to understand.

- Segmentation could become more advanced, since not all (small) MAPCAs were colored, and intrapulmonary connection could not be determined.

- Additional value to classify of (non-)communicating MAPCAs is low due to the lack of physiology/flow. In some cases, connection between PA and MAPCA was visualized, but only rarely.

- Threshold for surgeons is still too high due to the inability to load the images in the VR computer by themselves.

o Implementation of VR in preoperative planning is still wishful thinking in my opinion. However, it has some unique features, and due to the immersiveness, it forced me to examine the scan with more attention.

o Presence of a nasal gastric tube is a must, due to the consequence of the ability to segment and to visualize the esophagus, which provides important information

**Supplemental Figure A**


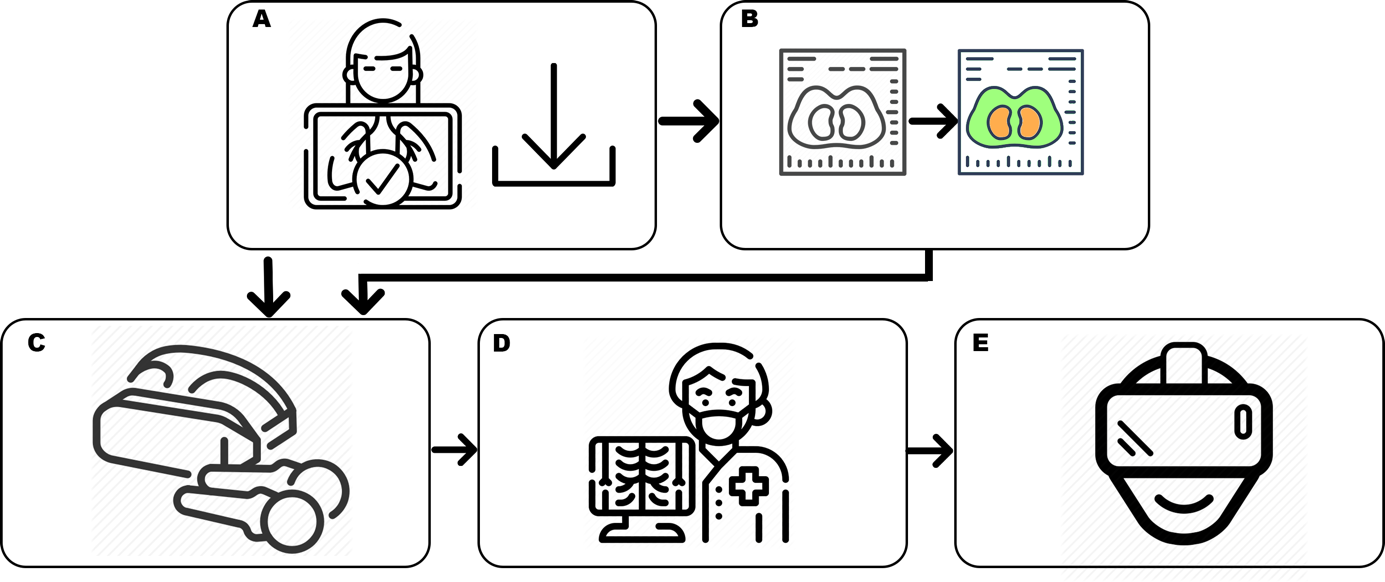

Supplement: sj-docx-1-pch-10.1177_21501351211045064 - Supplemental material for 3D Virtual Reality Imaging of Major Aortopulmonary Collateral Arteries: A Novel Diagnostic Modality [file sj-docx-1-pch-10.1177_21501351211045064.docx]
